# Subjective cognitive decline: The first clinical manifestation of Alzheimer's disease?

**DOI:** 10.1590/S1980-5764-2016DN1003002

**Published:** 2016

**Authors:** Adalberto Studart, Ricardo Nitrini

**Affiliations:** 1 Department of Neurology, Hospital das Clínicas da Faculdade de Medicina da Universidade de São Paulo, São Paulo SP, Brazil.

**Keywords:** subjective cognitive decline, dementia, Alzheimer's disease, biomarkers

## Abstract

**Background:**

Mild cognitive impairment is considered as the first clinical manifestation of Alzheimer's disease (AD), when the individual exhibits below performance on standardized neuropsychological tests. However, some subjects before having a lower performance on cognitive assessments already have a subjective memory complaint.

**Objective:**

A review about subjective cognitive decline, the association with AD biomarkers and risk of conversion to dementia.

**Methods:**

We performed a comprehensive non-systematic review on PubMed. The keywords used in the search were terms related to subjective cognitive decline.

**Results:**

Subjective cognitive decline is characterized by self-experience of deterioration in cognitive performance not detected objectively through formal neuropsychological testing. However, various terms and definitions have been used in the literature and the lack of a widely accepted concept hampers comparison of studies. Epidemiological data have shown that individuals with subjective cognitive decline are at increased risk of progression to AD dementia. In addition, there is evidence that this group has a higher prevalence of positive biomarkers for amyloidosis and neurodegeneration. However, Alzheimer's disease is not the only cause of subjective cognitive decline and various other conditions can be associated with subjective memory complaints, such as psychiatric disorders or normal aging. The features suggestive of a neurodegenerative disorder are: onset of decline within the last five years, age at onset above 60 years, associated concerns about decline and confirmation by an informant.

**Conclusion:**

These findings support the idea that subjective cognitive complaints may be an early clinical marker that precedes mild cognitive impairment due to Alzheimer's disease.

## RESUMO

**Introdução:**

O comprometimento cognitivo leve é considerado como a primeira manifestação clínica da doença de Alzheimer, quando o indivíduo exibe um desempenho abaixo para idade e escolaridade em testes neuropsicológicos padronizados. No entanto, alguns já apresentam uma queixa subjetiva de memória antes doprejuízo nas avaliações cognitivas.

**Objetivo:**

Fazer uma revisão sobre o declínio cognitivo subjetivo, a associação com biomarcadores da doença de Alzheimer e o risco de conversão para demência.

**Métodos:**

Realizou-se uma revisão não-sistemática no PubMed. As palavras-chave utilizadas na busca foram relacionadas ao declínio cognitivo subjetivo.

**Resultados:**

O declínio cognitivo subjetivo é caracterizada por uma autoexperiência da deterioração no desempenho cognitivo não detectado objetivamente por meio de testes neuropsicológicos formais. Todavia, vários termos e definições são utilizados na literatura e a falta de um conceito largamente aceito dificulta uma comparação. Os dados epidemiológicos mostram que indivíduos com declínio cognitivo subjetivo estão em maior risco de progressão para demência. Além disso, há evidências de que este grupo tem maior prevalência de biomarcadores positivos para amiloidose e neurodegeneração. Porém, a doença de Alzheimer não é a única causa e várias outras condições podem estar associadas, tais como distúrbios psiquiátricos ou o envelhecimento normal. As características sugestivas de uma doença neurodegenerativa são: início nos últimos cinco anos, início acima de 60 anos, estar preocupado com declínio e confirmação por um informante.

**Conclusão:**

Estes resultados suportam a ideia de que o declínio cognitivo subjetivo pode ser um marcador clínico precoce que precede comprometimento cognitivo leve devido à doença de Alzheimer.

## INTRODUCTION

Currently, the development of disease-modifying treatments for Alzheimer's disease (AD) targets stages of the disease prior to the dementia syndrome, in which neuronal damage is already irreversible. From the development of biomarkers that allow the detection of b-amyloid peptide, tau protein and neuronal injury, it is known that the AD pathology precedes the onset of dementia by many years.^1^ ^,^ ^2^ Consequently, identifying the first manifestation of AD with the aid of biomarkers can enable early diagnosis and therapeutic interventions.

The working group of the National Institute on Aging-Alzheimer's Association (NIA-AA) has produced new recommendations for the diagnosis of dementia due to Alzheimer's disease and proposed the concept of AD stages based on the model of the "amyloid cascade".^2^ According to this model, the disease runs in a continuous course from the preclinical phase (defined as absence of cognitive decline and presence of positive AD biomarkers) to the mild cognitive impairment stage (decline in at least one cognitive or behavioral domain without functional impairment and the presence of positive biomarker) through to the dementia phase due to Alzheimer's disease (impairment in at least two cognitive domains with functional decline).^1^ ^,^ ^3^ ^,^ ^4^ Also according to the NIA-AA, the preclinical phase begins with the cerebral amyloidosis stage (deposition of b-amyloid), followed by amyloidosis with neurodegeneration, and finally the subtle cognitive decline stage associated with positive AD biomarkers.^1^

This subtle cognitive decline is characterized by a self-experience of deterioration in cognitive performance not detected objectively through formal neuropsychological testing.^1^ ^,^ ^3^ ^,^ ^4^ Traditionally, the consensus considered mild cognitive impairment as the first clinical manifestation of the disease, when the individual exhibits below-average age-, gender- and education-adjusted performance on standardized neuropsychological tests.^3^ ^,^ ^4^ However, longitudinal studies, especially those using biomarkers, have shown that before having a lower performance on cognitive assessments, subjects already have a subjective memory complaint. The term "subjective cognitive decline" was created to describe this stage prior to mild cognitive impairment.^5^ ^,^ ^6^ This article entailed a non-systematic review on subjective cognitive decline, the association with AD biomarkers and risk of conversion to dementia. This knowledge is critical to understanding the early stages of the disease which can become targets of future earlier therapeutic interventions.

## METHODS

We performed a comprehensive literature non-systematic review on PubMed for references published between January 1990 and July 2016. The keywords used in the search were terms and words related to subjective cognitive decline: "subjective memory complaint", "self-reported memory complaint", "subjective cognitive impairment", "subjective cognitive concerns" , "Alzheimer's disease", "Alzheimer's biomarker". The search was restricted to articles written in English, Spanish and Portuguese language.

## RESULTS

Subjective cognitive decline: what is it and what is the risk? Various terms and definitions have been used in the literature, such as "subjective memory complaint", "self-reported memory complaint", "subjective cognitive impairment" and "subjective cognitive concerns".^5^ ^-^ ^12^ Consequently, the lack of a widely accepted concept, a "gold standard", makes it difficult to compare studies.^5^ ^,^ ^6^ A multicenter international working group (Subjective Cognitive Decline Initiative, SCD-I) was created with the objective to standardize terminology for studies and clinical trials.^5^ This group proposes the use of the term "subjective cognitive decline". "Subjective" because it refers to individual self-experience, regardless of objective performance on neuropsychological tests. The term "cognitive" rather than "memory" because the first symptoms of Alzheimer's disease are not restricted only to the memory domain. And finally, "decline" refers to the idea of progressive deterioration or a change from the previous level of functioning and not just an isolated complaint.^5^ ^,^ ^7^ Thus, the proposed criteria are: [1] self-experienced persistent decline in cognitive abilities compared with previously normal status and not related to an acute event; and [2] normal performance on standardized cognitive tests (for age, gender and education); while exclusion criteria are: [1] mild cognitive impairment or dementia diagnosis; and [2] decline explained by psychiatric disorders, neurological diseases (except Alzheimer's disease), other medical disorders, medication or other substance use.^5^

Several epidemiological studies have shown an increased risk of progression to AD dementia among individuals with subjective cognitive decline.^6^ ^-^ ^12^ However, these epidemiological studies differ in the method of evaluation of subjective complaints. While some studies were based on testimonies of participants, other applied standardized questionnaires (as discussed in the following section). Consequently, this difference of methodology impairs in part the comparison between these studies. Sixteen studies included in a systematic review showed a conversion risk to dementia or mild cognitive impairment that was 1.5-3 times higher in older age individuals with subjective cognitive complaints.^6^ Jessen et al., during a follow-up of six years, compared progression to dementia among normal controls, a subjective cognitive impairment group and another with mild cognitive impairment.^8^ The incidence of AD dementia in the group with subjective complaints was 1.5 times higher than in the control group. And when the subjects with memory complaints were concerned with their cognitive decline, the conversion rate to dementia was 2.44 times higher than in the control group, a value similar to that found in early mild cognitive impairment (defined as a performance on delayed recall between 1.5 SD and 1.0 SD below the normative mean).^8^ In another study involving the same cohort, the relationship between subjective cognitive decline and longitudinal performance on objective tests was examined over an 8-year period. The presence of subjective cognitive impairment at baseline predicted a more accelerated decline on episodic memory tests (immediate and delayed verbal recall) and more pronounced decline in the group of individuals with associated concerns.^9^

Reisberg et al. followed 213 individuals for about seven years and found that 54.2% of the group with subjective cognitive impairment declined to mild cognitive impairment or dementia, compared to 14.9% among normal controls (hazard ratio 4.5).^10^ Furthermore, subjects with complaints evolved more quickly to dementia than controls by an average of 3.5 years. Similar results were found in a Swedish study, a prospective follow-up for a median of ten years of a sample of 2043 individuals without dementia, which showed an incidence of dementia two to three times higher in the group with subjective cognitive impairment.^11^ In another study with a very long follow-up, 1107 older women (mean age: 70 years) were evaluated 18 years after the first visit where 52.8% of participants with subjective complaints progressed to dementia or mild cognitive impairment, while in the group without complaints the rate was 38%.^12^ Epidemiological studies have also used post-mortem pathological findings as an outcome. In one such study, subjective complaints were associated with higher amounts of neuritic amyloid plaques (but not neurofibrillary tangles) in the medial and neocortical regions of the temporal lobe in samples from autopsies.^13^

Some authors question whether the complaint of the individual would itself be sufficient to increase the risk of progression to dementia. This questioning arises from two observations: memory complaints are very prevalent in the elderly (and therefore can be nonspecific) and a high prevalence of lack of insight among patients with dementia exists. Consequently, some studies analyzed whether confirmation of cognitive decline by an informant increases the specificity and risk for progression to dementia.^14^ ^,^ ^15^ ^,^ ^16^ Gifford et al. observed that the isolated complaint from a patient had a conversion rate of 2.1; similar to the risk of progression when only the informant reports the complaint. On the other hand, if both the individual and informant report cognitive decline, the risk rises to 4.2.^14^

However, akin to individuals with mild cognitive impairment, many with subjective complaints may remain stable or even show improvement in cognition. Alzheimer's disease is not the only cause of subjective cognitive decline and various other conditions can be associated with subjective memory complaints, such as normal aging, personality traits, psychiatric disorders and use of psychiatric drugs.^5^ ^,^ ^7^ Within this heterogeneous group, how can individuals who will progress to mild cognitive impairment or dementia be identified? And what are the features of subjective cognitive complaint that aggregate this risk of cognitive decline? In other words, how can we determine whether an elderly individual with subjective cognitive decline presents an underlying preclinical Alzheimer's disease and not another cause, such as a depressive disorder?

Subjective cognitive decline: how can it be identified? Although several studies have evaluated subjective cognitive decline and its risk of progression to dementia, there is no standard on how the evaluation should be carried out. Ideally, the assessment of subjective cognitive decline in any study must provide balance and not be too sensitive (with high false positive rates) or very specific (and therefore too restrictive).^5^ ^,^ ^17^ How can we objectively evaluate a subjective complaint? 

Several questionnaires and scales have been developed and applied in clinical and epidemiological studies. The most frequent self-reported measures include: Questionnaire AgeCoDe Study,^18^ Everyday Cognition scale (E-cog),^19^ Memory Functioning Questionnaire (MFQ),^20^ Subjective memory decline scale (SMDS),^21^ Memory complaint questionnaire (MAC-Q),^22^ Memory failures everyday - 30 (MFE - 30),^23^ Structured Telephone Interview for Dementia Assessment (STIDA),^24^ Informant Questionnaire on Cognitive Decline in the Elderly (IQCODE)^25^ and Subjective Memory Complaints (SMC).^26^ These differ in mode of administration, number of items, timeframe referenced by items, cognitive domains reported as complaints and degree of severity. Some self-reported measures explore only memory complaints, while others evaluate decline in other cognitive abilities. Even among the scales that include memory complaints only, some are generic issues while other measures evaluate specific everyday situations. Another difference is a large variability among questionnaires in relation to response timeframe and subjective perception.. Some of these instruments compare current cognitive performance with a few weeks or months ago, while others compare with several years ago or even when younger.^17^

As previously mentioned, not all subjective memory complaint is due to pre-clinical Alzheimer's disease. Of other causes, mental disorders are among the most often associated and therefore the evaluation of patients with subjective memory complaint must include a neuropsychiatric inventory. However, even excluding patients who meet criteria for a major psychiatric disorder, symptoms of depression and anxiety or morbid personality traits are fairly frequently observed in epidemiological studies.^8^ ^,^ ^12^ ^,^ ^15^ Thus, in clinical practice, how can it be distinguished whether a cognitive complaint is due to a mental disorder or Alzheimer's disease in the preclinical stage? For this purpose, some authors have coined the term "subjective cognitive decline plus" whose features increase the likelihood of an underlying neurodegenerative disorder.^5^ ^,^ ^6^ For example, a decline noticed by the patient within a five-year interval increases the probability of preclinical AD. In contrast, complaints in a patient with a history of many years most likely have causes other than AD. Age of onset is also an important feature to distinguish neurological and psychiatric causes. It is more likely for an elderly person than a young adult to have subjective decline due to Alzheimer's disease. As discussed in the studies cited above, associated concerns with complaint and confirmation of decline by an informant are features that suggest a neurodegenerative cause. In summary, elderly with increased risk of conversion to dementia are those with progressive memory complaint within the last five years, concerns about decline, and whose family members confirm this decline. 

Although subjective cognitive decline is defined as a complaint without detectable impairment by standardized neuropsychological tests, a new generation of episodic memory tests has shown utility for the diagnosis of Alzheimer's preclinical disease with a good correlation with subjective complaint.^27^ ^-^ ^29^ For example, a task of Short-Term Memory Binding (STMB) has recently been validated and shown to be more sensitive than traditional neuropsychological evaluations in individuals with subjective cognitive decline for diagnosing the pre-clinical stage of Alzheimer's disease.^27^ Other tests developed, which have also shown good correlation with subjective cognitive impairment, were the Name Face Associative Memory Exam (FNAME)^28^ and evaluation of prospective memory.^29^ Therefore, these are useful tools in the clinical evaluation of patients with subjective cognitive decline, which increases the specificity of the complaint as a marker of preclinical Alzheimer's disease.

Subjective cognitive decline studies in the Brazilian population. Although an increasingly discussed topic in Cognitive Neurology, there are few studies involving the Brazilian population.^30^ ^-^ ^35^ Moreover, to date, there are no published Brazilian studies involving AD biomarkers in patients with subjective cognitive decline. 

In a community-based study, seventy-one healthy older adults were asked if they had memory complaints, filled out the Memory Complaint Questionnaire (MAC-Q) and underwent a formal test of episodic memory (Rey Auditory Verbal Learning Test - RAVLT). Spontaneous complaints were associated with poor performance on the RAVLT, but not on the MAC-Q.^33^ Another study analyzed the presence of memory complaint in 163 subjects without dementia in a forest reserve in the Brazilian Amazon. The study volunteers were submitted to the Mini-Mental State Examination (MMSE), delayed recall from the Brief Cognitive Battery and a questionnaire assessing psychiatric symptoms. Results revealed a positive correlation between the presence of psychiatric symptoms and lower MMSE scores but not delayed recall test scores.^34^

On the other hand, some studies have shown no correlation between worse performance on cognitive tests and the presence of subjective decline.^35^ ^,^ ^36^ In one investigation, two groups were compared: one formed by community elders with complaints and another consisting of institutionalized elderly without complaints. No differences were observed between the groups in performance on neuropsychological assessment.^35^ Similar findings were also obtained in a sample of normal elderly (caregivers of patients with dementia), where there was no statistically significant correlation between subjective complaints and objective cognitive assessment.^36^

Alzheimer's disease biomarkers in subjective cognitive decline. In recent years, several studies (such as the "Alzheimer's Disease Neuroimaging Initiative - ADNI" and "Australian Imaging Biomarkers and Lifestyle study - AIBL") have been investigating the correlation *in vivo* of pathologic findings (using biomarkers) with clinical manifestations of Alzheimer's disease.^1^ ^,^ ^7^ ^,^ ^38^ ^,^ ^39^ Patholo gical processes most widely investigated through biomarkers are: brain amyloidosis (by decreased b-amyloid peptide in cerebrospinal fluid and PET-CT with positive ligands for b-amyloid - for example Pittsburgh compound B, PiB) and neurodegeneration (by elevated total tau and phosphorylated tau proteins in the cerebrospinal fluid, cortical atrophy on structural magnetic resonance imaging, MRI, and decreased glycolytic metabolism in 18F-fluorodeoxyglucose PET-CT).^1^ ^,^ ^2^ While there are studies with evidence of increased frequency of amyloidosis and neurodegeneration associated with aging in cognitively normal patients,^39^ ^-^ ^42^ the presence of amyloidosis in normal elderly is associated with higher rates of progression to mild cognitive impairment and dementia.^39^ ^,^ ^42^ ^-^ ^45^ Consequently, the use of biomarkers attempts to resolve the above question by identifying which individuals with subjective cognitive decline have a higher risk of progressing to an AD dementia stage. Structural MRI, PET-CT with positive ligands for b-amyloid and 18F-fluorodeoxyglucose PET-CT (FDG-PET), are methods that both determine the stage of progression of the disease as well as elucidate the anatomical extent of the disease.^46^ ^-^ ^51^

Perrotin et al. compared two groups of normal elderly, one with positive PiB PET-CT and another with negative PiB PET-CT, for subjective cognition and performance on a neuropsychological evaluation.^47^ The positive PiB group had a higher frequency of subjective cognitive complaints and worse performance in episodic memory tests. In addition, some anatomical regions of interest showed a positive correlation between increased uptake of radiotracer for amyloid and subjective decline (right medial frontal/anterior cingulate cortex and right precuneus/posterior cingulate cortex).^47^ Amariglio et al. applied three subjective cognition questionnaires and a neuropsychological battery in a group of 130 elderly who underwent a PiB-PET. A positive correlation between memory complaints and cortical PiB binding was found. On the other hand, performance on tests of episodic memory and executive functions and cortical PiB binding did not show a statistically significant correlation.^48^ The same research group also used PiB-PET, FDG-PET and structural MRI, classifying normal elderly patients into four groups according to status of biomarkers for amyloidosis (Ab) and neurodegeneration (nd): negative biomarker (Ab- / ND-), amyloidosis alone (Ab b+ / ND-), amyloidosis plus neurodegeneration (Ab+ / NA+) and suspected non-Alzheimer disease pathophysiology (Ab- / ND+).^35^ Participants filled out a questionnaire for subjective cognitive complaints and the presence of biomarkers correlated with more complaints, especially in the groups with positive Ab.^49^ Similar results were found in a recent longitudinal study.^50^ Fifty-eight normal older adults with positive b-amyloid-PET answered a questionnaire of subjective memory decline and underwent neuropsychological assessment. After a follow-up of three years, it was found that individuals with complaints had a higher rate of progression to mild cognitive impairment or dementia (hazard ratio of 5.1) compared to those without complaints. However, two groups did not differ in decline on formal tests of episodic memory. Moreover, the group with complaints had a higher incidence of depressive symptoms and lower left hippocampus volume.^50^ However, some studies have failed to demonstrate the relationship between the presence of biomarkers and subjective decline. A prospective study of 289 healthy elderly found no differences between PiB-PET status and presence of subjective memory complaint, although a group with positive PiB-PET showed a moderate decline in working memory and learning.^51^ Recently, the development of a PET tracer with high affinity for tau protein has allowed to understand the distribution of tau aggregates in vivo.^52^ ^,^ ^53^ However, there are still no published studies of tau imaging in normal individuals with subjective memory complaints.

Evidence also points to a correlation between subjective cognitive decline and cortical atrophy on structural MRI, especially in commonly vulnerable regions for Alzheimer disease.^54^ ^-^ ^57^ In one such study, two hundred and sixty-one healthy middle-age adults were asked whether they had a memory problem. The group with cognitive complaints had significant cortical thinning in the entorhinal, fusiform, posterior cingulate and posterior parietal cortices, as well as reduced amygdala volume.^54^ Additionally, this same group showed worse performance on memory tests, even within the normal range. A pattern of gray matter atrophy, similar to that found in Alzheimer's disease, has been found in a group of 226 subjects with subjective memory disorders in a German study.^55^ A cross-sectional study compared hippocampal volume in 47 healthy elderly with memory complaints and 48 normal controls and measured the plasma levels of beta amyloid. Those with subjective complaints showed lower volume in CA1, CA2, dentate gyrus and the molecular layer and had higher levels of beta amyloid.^56^ Other neuroimaging methods have also been exploited by several studies on subjective cognitive decline, such as functional magnetic resonance imaging (fMRI) and MRI with diffusion tensor imaging (DTI).^58^ ^,^ ^59^ In one fMRI study, there was less activation of the right hippocampus in patients with subjective decline during the course of episodic memory testing, despite the fact that test performance was within the normal range.^58^ And a Japanese study examined the association of white matter connectivity (by performing MRI-DTI) and amyloid deposition (PiB-PET) and compared two groups (one with subjective cognitive impairment and another without complaint). In the end, it was found that individuals with subjective cognitive impairment had reduced functional connectivity between retrosplenial cortex and anterior medial cortical structures.^59^

Similarly to imaging methods, studies of b-amyloid peptide, total tau and phosphorylated tau proteins in cerebrospinal fluid (CSF) are widely used in investigations of subjective cognitive decline.^60^ ^-^ ^64^ In a multicenter cohort, the frequency of individuals with subjective complaint and positive AD biomarkers in CSF was superior to the control group (52% vs. 31%).^60^ A Spanish longitudinal study followed 149 subjects without dementia (but with subjective cognitive decline or mild cognitive impairment) for five years and found that only 15% of those with pathological CSF remained free of AD dementia. The odds ratio for conversion to dementia was 27.1, demonstrating that an abnormal AD CSF biomarker profile is a powerful predictor for cognitive and functional decline.^61^ In a Dutch longitudinal study, a sample of 127 volunteers with subjective decline was followed by two years after collection of CSF biomarkers. The study found that a decrease in Ab CSF was the strongest predictor of progression to mild cognitive impairment and dementia (odds ratio 16).^61^

In addition to the biomarkers, AD has many other risk factors, but none is better established than the e4 allele of apolipoprotein E (APOE). There is ample evidence that individuals with subjective cognitive decline have a greater frequency of expression of the allele, especially among those with positive biomarkers.^65^ ^,^ ^66^ Samieri et al. compared subjective cognitive decline among APOE e4 carriers and non-carriers for over six years. The authors concluded that APOE e4 carriers evolved with faster memory decline.^65^ In a more recent study, normal elderly with memory complaints were also grouped into APOE e4 carriers and non-carriers and underwent amyloid PET, FDG PET, structural MRI and CSF biomarker testing. The APOE e4 carriers had changes in amyloid PET and CSF biomarkers. Therefore, these results indicate an association between the APOE e4 allele and AD biomarkers in older adults with memory complaints.^66^

## CONCLUSIONS

Several studies have shown that individuals with subjective cognitive decline are at increased risk of progression to AD dementia. According to epidemiological data, the features which increase the likelihood of conversion are: onset of decline within the last five years, age at onset above 60 years, associated concerns about decline and confirmation by an informant. In addition, there is evidence that this group has a higher prevalence of positive biomarkers for amyloidosis and neurodegeneration. Consequently, these findings support the idea that subjective cognitive complaints may be an early clinical marker of pathology and help further understanding on the natural history of Alzheimer's disease from pre-dementia stages. However, due to lack of consensus on how to define and assess subjective cognitive decline, it is still unclear which characteristics of subjective cognitive decline suggest the preclinical AD stage.

## References

[1] Sperling RA, Aisen S, Beckett LA (2011). Toward defining the preclinical stages of Alzheimer's disease
Recommendations from the National Institute on Aging Alzheimer's Association
workgroups on diagnostic guidelines for Alzheimer's disease. Alzheimers Dement.

[2] Jack CR, Knopman DS, Jagust WJ (2010). Hypothetical model of dynamic biomarkers of the Alzheimer's
pathological cascade. Lancet Neurol.

[3] McKhann GM, Knopman DS, Chertkow H (2011). The diagnosis of dementia due to Alzheimer's disease: Recommendations
from the National Institute on Aging-Alzheimer's Association workgroups on
diagnostic guidelines for Alzheimer's disease. Alzheimers Dement.

[4] Frota NAF, Nitrini R, Damasceno BP (2011). Critérios para o diagnóstico de doença de Alzheimer. Dement Neuropsychol.

[5] Jessen F, Amariglio RE, Boxtel van (2014). A conceptual framework for research on subjective cognitive decline in
preclinical Alzheimer's disease. Alzheimers Dement.

[6] Mendonça MD, Alves L, Bugalho P (2016). From Subjective Cognitive Complaints to Dementia Who is at Risk?: A
Systematic Review. Am J Alzheimers Dis Other Demen.

[7] Jessen F (2014). Subjective and objective cognitive decline at the pre-dementia stage
of Alzheimer's disease. Eur Arch Psychiatry Clin Neurosci.

[8] Jessen F, Wolfsgruber S, Wiese B (2014). AD dementia risk in late MCI, in early MCI, and in subjective memory
impairment. Alzheimers Dement.

[9] Koppara A, Wagner M, Lange C (2015). Cognitive performance before and after the onset of subjective
cognitive decline in old age. Alzheimers Dement (Amst).

[10] Reisberg B, Shulman MB, Torossian C (2010). Outcome over seven years of healthy adults with and without subjective
cognitive impairment. Alzheimers Dement.

[11] Rönnlund M, Sundström A, Adolfsson R (2015). Subjective memory impairment in older adults predicts future dementia
independent of baseline memory performance Evidence from the Betula prospective
cohort study. Alzheimers Dement.

[12] Kaup AR, Nettiksimmons J, LeBlanc ES (2015). Memory complaints and risk of cognitive impairment after nearly 2
decades among older women. Neurology.

[13] Kryscio RJ, Abner EL, Cooper GE (2014). Self-reported memory complaints implications from a longitudinal
cohort with autopsies. Neurology.

[14] Gifford KA, Liu D, Lu Z (2014). The source of cognitive complaints predicts diagnostic conversion
differentially among nondemented older adults. Alzheimers Dement.

[15] Buckley R, Saling M, Ellis K (2015). Self and informant memory concerns align in healthy memory complainers
and in early stages of mild cognitive impairment but separate with increasing
cognitive impairment. Age Ageing.

[16] Caselli RJ, Chen K, Locke DE (2014). Subjective cognitive decline self and informant
comparisons. Alzheimers Dement.

[17] Rabin LA, Smart CM, Crane PK (2015). Subjective Cognitive Decline in Older Adults: An Overview of
Self-Report Measures Used Across 19 International Research Studies. J Alzheimers Dis.

[18] Jessen F, Wiese B, Cvetanovska G (2007). Patterns of subjective memory impairment in the elderly Association
with memory performance. Psychol Med.

[19] Farias ST, Mungas D, Reed BR (2008). The measurement of everyday cognition (ECog) Scale development and
psychometric properties. Neuropsychology.

[20] Gilewski MJ, Zelinski EM, Schaie KW (1990). The memory functioning questionnaire for assessment of memory
complaints in adulthood and old age. Psychol Aging.

[21] Jorm AF (1994). A short form of the Informant Questionnaire on Cognitive Decline in
the Elderly (IQCODE) Development and cross-validation. Psychol Med.

[22] Crook THE, Feher EP, Larrabee GJ (1992). Assessment of memory complaint in age-associated memory impairment The
MAC-Q. Int Psychogeriatr.

[23] Lozoya-Delgado P, Ruiz-Sanchez de Leon JM, Pedrero-Perez EJ (2012). Validación de un cuestionario de quejas cognitivas para adultos
jóvenes Relación entre las quejas subjetivas de memoria, la sintomatología
prefrontal y el estrés percibido. Rev Neurol.

[24] Go RCP, Duke LW, Harrell LE (1997). Development and validation of a Structured Telephone Interview for
Dementia Assessment (STIDA) The NIMH Genetics Initiative. J Geriatr Psychiatry Neurol.

[25] Jorm AF, Christensen H, Korten AE, Jacomb PA, Henderson AS (2001). Memory complaints as a precursor of memory impairment in older people
A longitudinal analysis over 7-8 years. Psychol Med.

[26] Vogel A, Salem LC, Andersen BB, Waldemar G (2016). Differences in quantitative methods for measuring subjective cognitive
decline - results from a prospective memory clinic study. Int Psychogeriatr.

[27] Koppara A, Frommann I, Polcher A (2015). Feature Binding Deficits in Subjective Cognitive Decline and in Mild
Cognitive Impairment. J Alzheimers Dis.

[28] Alegret M, Rodríguez O, Espinosa A (2015). Concordance between Subjective and Objective Memory Impairment in
Volunteer Subjects. J Alzheimers Dis.

[29] Hsu YH, Huang CF, Tu MC (2015). Prospective memory in subjective cognitive decline a preliminary study
on the role of early cognitive marker in dementia. Alzheimer Dis Assoc Disord.

[30] Almeida OP (1998). Memory complaints and the diagnosis of dementia. Arq Neuropsiquiatr.

[31] Minett TS, Da Silva RV, Ortiz KZ, Bertolucci PH (2008). Subjective memory complaints in an elderly sample a cross-sectional
study. Int J Geriatr Psychiatry.

[32] Brigola AG, Manzini CSS, Oliveira GBS (2015). Subjective memory complaints associated with depression and cognitive
impairment in the elderly A systematic review. Dement Neuropsychol.

[33] Mattos P, Lino V, Rizo L (2003). Memory complaints and test performance in healthy elderly
persons. Arq Neuropsiquiatr.

[34] Brucki SM, Nitrini R (2009). Subjective memory impairment in a rural population with low education
in the Amazon rainforest an exploratory study. Int Psychogeriatr.

[35] Aguiar ACPO, Ribeiro MI, Jacinto AF (2010). Subjective memory complaints in the elderly may be related to factors
other than cognitive déficit. Dement Neuropsychol.

[36] Caramelli P, Beato RG (2008). Subjective memory complaints and cognitive performance in a sample of
healthy elderly. Dement Neuropsychol.

[37] Vale FAC, Balieiro-Jr AP, Silva-Filho JH (2012). Memory complaint scale (MCS) Proposed tool for active systematic
search. Dement Neuropsychol.

[38] Ellis KA, Rowe CC, Villemagne VL (2010). Addressing population aging and Alzheimer's disease through the
Australian imaging biomarkers and lifestyle study collaboration with the
Alzheimer's Disease Neuroimaging Initiative. Alzheimers Dement.

[39] Landau SM, Mintun MA, Joshi AD (2012). Amyloid deposition, hypometabolism, and longitudinal cognitive
decline. Ann Neurol.

[40] Jack CR, Wiste HJ, Weigand SD (2014). Age-specific population frequencies of cerebral b-amyloidosis and
neurodegeneration among people with normal cognitive function aged 50-89 years a
cross-sectional study. Lancet Neurol.

[41] Toledo JB, Zetterberg H, van Harten AC (2015). Alzheimer's disease cerebrospinal fluid biomarker in cognitively
normal subjects. Brain.

[42] Snitz BE, Weissfeld LA, Lopez OL (2013). Cognitive trajectories associated with b-amyloid deposition in the
oldest-old without dementia. Neurology.

[43] Villain N, Chételat G, Grassiot B (2012). Regional dynamics of amyloid-b deposition in healthy elderly, mild
cognitive impairment and Alzheimer's disease a voxelwise PiB-PET longitudinal
study. Brain.

[44] Resnick SM, Sojkova J, Zhou Y (2010). Longitudinal cognitive decline is associated with fibrillar
amyloid-beta measured by [11C]PiB. Neurology.

[45] Doré V, Villemagne VL, Bourgeat P (2013l). Cross-sectional and longitudinal analysis of the relationship between
Ab deposition, cortical thickness, and memory in cognitively unimpaired
individuals and in Alzheimer disease. JAMA Neurol.

[46] Snitz BE, Lopez OL, McDade E (2015). Amyloid-b Imaging in Older Adults Presenting to a Memory Clinic with
Subjective Cognitive Decline A Pilot Study. J Alzheimers Dis.

[47] Perrotin A, Mormino EC, Madison CM (2012). Subjective cognition and amyloid deposition imaging a Pittsburgh
Compound B positron emission tomography study in normal elderly
individuals. Arch Neurol.

[48] Amariglio RE, Becker JA, Carmasin J (2012). Subjective cognitive complaints and amyloid burden in cognitively
normal older individuals. Neuropsychologia.

[49] Amariglio RE, Mormino EC, Pietras AC (2015). Subjective cognitive concerns, amyloid-b, and neurodegeneration in
clinically normal elderly. Neurology.

[50] Buckley RF, Maruff P, Ames D (2016). Subjective memory decline predicts greater rates of clinical
progression in preclinical Alzheimer's disease. Alzheimers Dement.

[51] Hollands S, Lim YY, Buckley R (2015). Amyloid-b related memory decline is not associated with subjective or
informant rated cognitive impairment in healthy adults. J Alzheimers Dis.

[52] Ossenkoppele R, Schonhaut DR, Schöll M (2016). Tau PET patterns mirror clinical and neuroanatomical variability in
Alzheimer's disease. Brain.

[53] Villemagne VL, Fodero-Tavoletti MT, Masters CL, Rowe CC (2015). Tau imaging early progress and future directions. Lancet Neurol.

[54] Schultz SA, Oh JM, Koscik RL (2015). Subjective memory complaints, cortical thinning, and cognitive
dysfunction in middle-aged adults at risk for AD. Alzheimers Dement (Amst).

[55] Peter J, Scheef L, Abdulkadir A (2014). Gray matter atrophy pattern in elderly with subjective memory
impairment. Alzheimers Dement.

[56] Cantero JL, Iglesias JE, Van Leemput K, Atienza M (2016). Regional Hippocampal Atrophy and Higher Levels of Plasma Amyloid-Beta
Are Associated With Subjective Memory Complaints in Nondemented Elderly
Subjects. J Gerontol A Biol Sci Med Sci.

[57] Jessen F, Feyen L, Freymann K (2006). Volume reduction of the entorhinal cortex in subjective memory
impairment. Neurobiol Aging.

[58] Erk S, Spottke A, Meisen A (2011). Evidence of neuronal compensation during episodic memory in subjective
memory impairment. Arch Gen Psychiatry.

[59] Yasuno F, Kazui H, Yamamoto A (2015). Resting-state synchrony between the retrosplenial cortex and anterior
medial cortical structures relates to memory complaints in subjective cognitive
impairment. Neurobiol Aging.

[60] Visser PJ, Verhey F, Knol DL (2009). Prevalence and prognostic value of CSF markers of Alzheimer's disease
pathology in patients with subjective cognitive impairment or mild cognitive
impairment in the DESCRIPA study a prospective cohort study. Lancet Neurol.

[61] van Harten AC, Visser PJ, Pijnenburg YA (2013). Cerebrospinal fluid Ab42 is the best predictor of clinical progression
in patients with subjective complaints. Alzheimers Dement.

[62] van Harten AC, Smits LL, Teunissen CE (2013). Preclinical AD predicts decline in memory and executive functions in
subjective complaints. Neurology.

[63] Wolfsgruber S, Jessen F, Koppara A (2015). Subjective cognitive decline is related to CSF biomarkers of AD in
patients with MCI. Neurology.

[64] Sierra-Rio A, Balasa M, Olives J (2016). Cerebrospinal Fluid Biomarkers Predict Clinical Evolution in Patients
with Subjective Cognitive Decline and Mild Cognitive Impairment. Neurodegener Dis.

[65] Samieri C, Proust-Lima C, M Glymour M (2014). Subjective cognitive concerns, episodic memory, and the APOE e4
allele. Alzheimers Dement.

[66] Risacher SL, Kim S, Nho K (2015). APOE effect on Alzheimer's disease biomarkers in older adults with
significant memory concern. Alzheimers Dement.

